# Topographical anatomy of the albino rat’s ischiotrochanteric muscle group

**DOI:** 10.1038/s41598-022-23379-3

**Published:** 2022-11-03

**Authors:** Bettina Pretterklieber, Michael L. Pretterklieber, Katharina Kerschan-Schindl

**Affiliations:** 1 Macroscopic and Clinical Anatomy, Gottfried Schatz Research Center, Medical University of Graz, Harrachgasse 21, 8010 Graz, Austria; 2 Division of Anatomy, Center for Anatomy and Cell Biology, Medical University of Vienna, Vienna, Austria; 3 Department of Physical Medicine, Rehabilitation and Occupational Medicine, Medical University of Vienna, Vienna, Austria

**Keywords:** Skeletal muscle, Translational research

## Abstract

The obturator internus, gemelli, and quadratus femoris muscles are thought to be postural muscles. Thus, they are in the focus of research. Although these muscles are described in other species, detailed descriptions are still lacking for the albino rat. We hypothesized that the rat’s ischiotrochanteric muscle group is comparable to that of humans. We aimed to provide a detailed description, and to compare the rat’s condition with other species including humans. Both hind limbs of 30 adult male formalin-fixed albino rats were carefully dissected and photo documented. Our results were then compared with data for other species and descriptions of human anatomy. The gemellus muscle was one single muscle mass, originating from the lesser sciatic notch and an unnamed groove on the dorsal aspect of the ischium. The obturator internus muscle arose from the inner aspect of the tabula of ischium. Both muscles formed a continuum and inserted as one complex on the medial aspect of the greater trochanter. The quadratus femoris muscle originated from the outer aspect of the tabula of ischium and inserted on the distal portion of the intertrochanteric crest, and the dorsal aspect of the lesser trochanter. Despite minor differences, the topographical conditions of these muscles are comparable between rats and other mammals including humans.

## Introduction

Phylogenetically and ontogenetically, in all mammals including humans, the ischiotrochanteric muscle group includes the quadratus femoris muscle, the obturator internus muscle, and the gemelli muscles. These muscles are innervated by two nerves arising variably from the sacral plexus, the nerve to quadratus femoris muscle, and the nerve to obturator internus muscle^1–5^. The muscles of the ischiotrochanteric group, together with the piriformis muscle and the obturator externus muscle, form the short lateral (external) rotators of the hip joint, often referred to as the "rotator cuff of the hip joint"^6^. These muscles are thought to be important as postural muscles, more than as prime movers^7^. For this reason, they are also the focus of research^8–11^. Animal models are often used for this purpose^12,13^.

Although these muscles are partly well described in humans and primates^6–8,14,15^, there are no sufficiently precise descriptions for the albino rat, which is after all used as a frequent animal model for basic^16^ and translational research^17^. Books on the anatomy of the laboratory rat^18,19^ outline this subject only cursorily or erroneously and are therefore neither useful for comparison with other species (including humans) nor for exact dissection. On the other hand, there are no descriptions of rodents even in detailed books of comparative anatomy^4,20^, which would be helpful for exact dissection of the hip and thigh regions in the rat. According to former comparative anatomists, the ischiotrochanteric muscle group is said to hardly vary within mammals, which makes them well comparable between species^4,21^.

We hypothesize that the topography of the rat ischiotrochanteric muscle group is also comparable to that of other mammals including humans. To verify this, the first goal was to provide a complete and accurate description and a topographical dissection guide of the muscles of the ischiotrochanteric group in the albino rat. The second aim was to discuss the similarities and differences of the condition found in albino rats and other mammals, including humans from a pure topographical point of view.

## Methods

To clarify the general arrangement of the muscles of the ischiotrochanteric group, both hind limbs of 30 adult (12–14 weeks old) male albino rats (24 Sprague-Dawley, 6 Wistar) have been carefully dissected. The rats had been used for other studies not involving the regions of interest and had been sacrificed before being placed to our disposal. Therefore, according to Austrian law, no approval of the local animal research ethics committee was necessary. The trunk and hind limbs were skinned; the abdominal and pelvic organs including the external genitals were carefully removed. Therefore, the branches of the pudendal nerve ended free during further dissection. The rats were fixed by immersion with a 2% solution of non-buffered formalin for some weeks. Following fixation, the specimens were stored in a low-percent solution of phenol.

Dissections were performed using a magnification lens. Exact description of the systematical and topographical anatomy was done by careful stratigraphic dissection similar done to individualize hip muscles in human specimens. As the establishment of a dissection guide was one of the aims of this study, the detailed dissection protocol is given in the results section. Photographs were taken using a digital reflex camera (Canon EOS 5D Mark II, Canon Inc., Tokyo, Japan). All terms used in this study are following the English version of the Nomina anatomica veterinaria^22,23^. In absence of official terms given by the veterinary anatomy, the terms have been used according to the English version of the Terminologia anatomica^24–26^. In exceptional instances, other definitions were applied.

**Figure 1:**
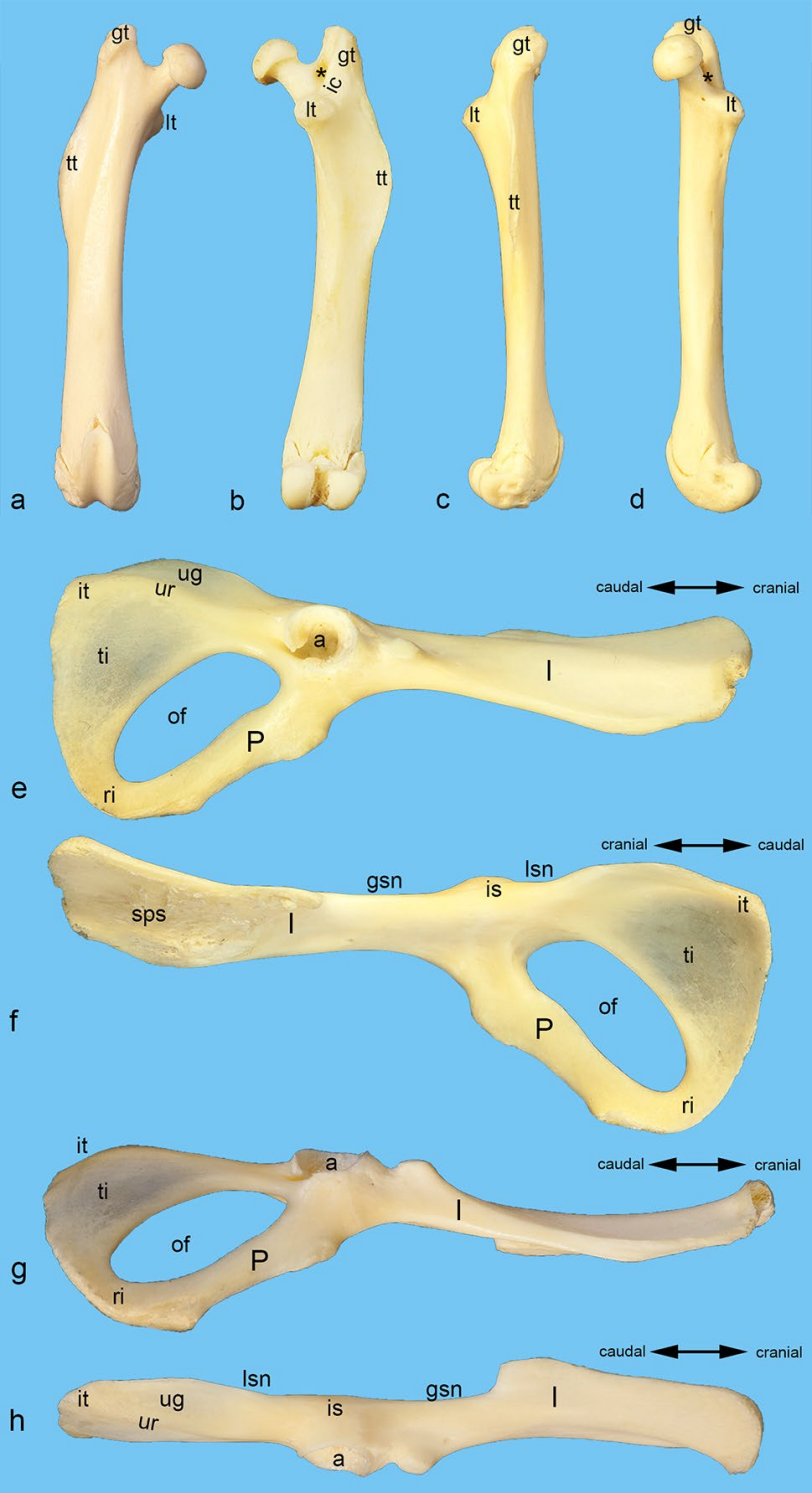
Bony structures important for this study. Macerated bones from a male albino rat were used to provide an overview of the bony structures important for this study. Isolated right femur from (**a**) the cranial, (**b**) the caudal, (**c**) the lateral, and (**d**) the medial aspect. Isolated right hip bone from (**e**) the lateral, (**f**) the medial, (**g**) the ventral, and (**h**) the dorsal aspect. a = acetabulum, gsn = greater sciatic notch, gt = greater trochanter, I = ilium, ic = intertrochanteric crest, it = ischial tuber, is = ischial spine, lsn = lesser sciatic notch, lt = lesser trochanter, of = obturator foramen, P = pubis, ri = ramus of ischium, sps = sacropelvic surface (auricular surface and iliac tuberosity), ti = tabula of ischium, tt = third trochanter, ug = unnamed groove dorsal on the ischium, ur = unnamed ridge dorsal on the ischium, asterisk = trochanteric fossa.

**Figure 2:**
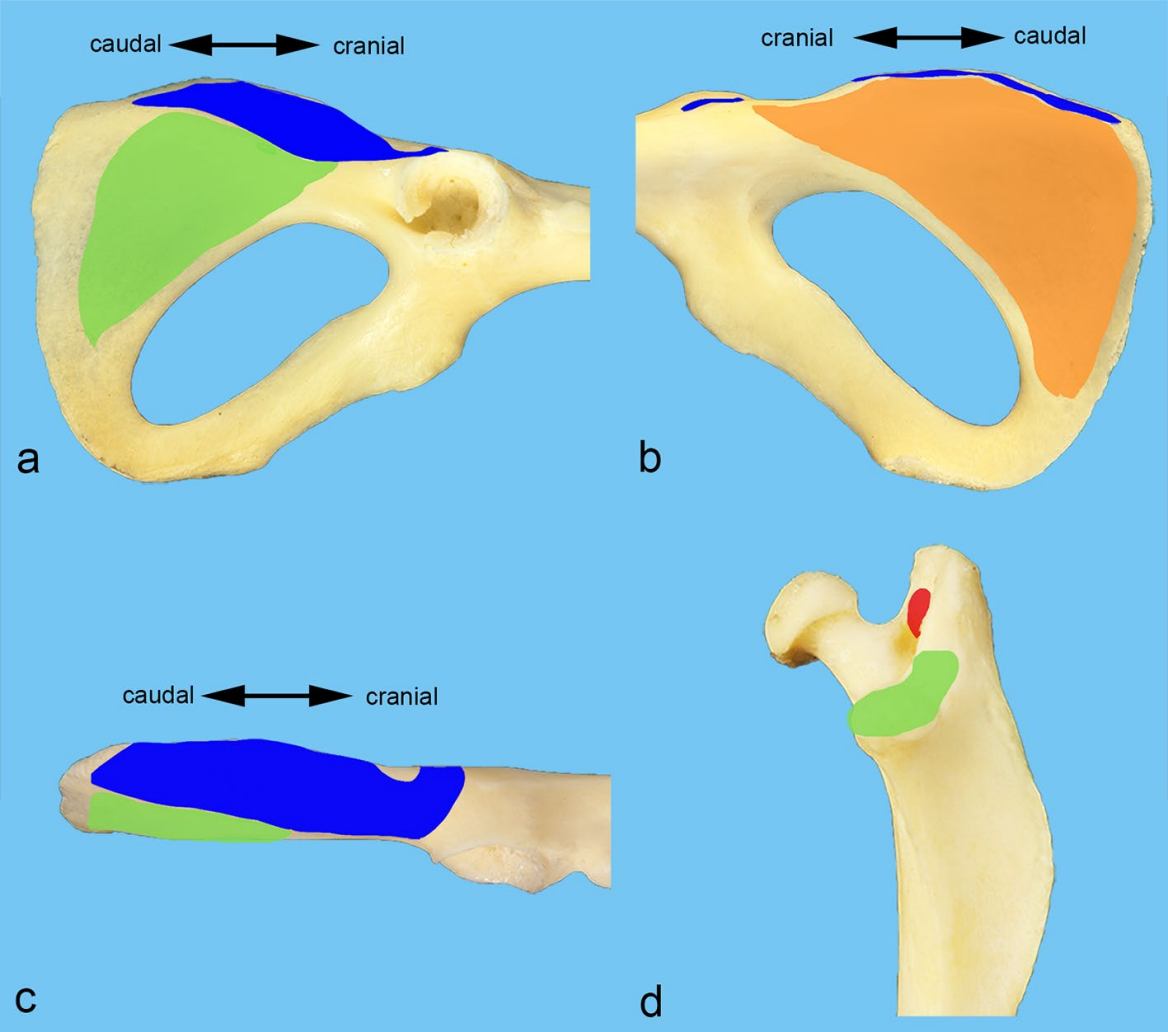
Muscle attachments of the ischiotrochanteric group. Origin and insertion areas of the muscles of the ischiotrochanteric group are color-coded on the pictures of isolated macerated bones from a male albino rat. The caudal half of the hip bone is shown (**a**) from lateral, (**b**) from medial, and (**c**) from dorsal. (**d**) The proximal end of the femur is shown from caudal. Color key: blue—gemellus muscle, orange—obturator internus muscle, red—tendon of biceps coxae muscle, green—quadratus femoris muscle.

Furthermore, macerated bones from one additional male albino rat were used to provide an overview of the bony structures important for this study (Fig. 1). Using Adobe Photoshop CS6, the origin and attachment areas of all the muscles examined in this study were color-coded in some of these photos (Fig. 2).

General comparative and veterinary textbooks and studies were used to compare rat hip muscles with other mammals. The comparison with human anatomy is mainly based on detailed human anatomical textbooks, some studies concerning the anatomy of the hip muscles and our own years of experience in the dissecting room. The respective references are given with the corresponding statements in the discussion.

In order to deduce the main functions in the hip joint of the studied muscles, the muscle courses were simulated on a macerated rat skeleton. The main axes for the joint were determined according to arthrokinematics. The possible movements produced by the muscles were derived based on the generally applied rules of the law of levers.

## Results

The systematic details of the investigated muscles are summarized in Table 1. Within this muscle group, there were no remarkable variations. Only, the exact origin of the nerve to obturator internus muscle was subject to small variations concerning its exact origin. This was due to the somewhat variable formation of the sacral plexus and its branches.

**Table 1:** Systematic anatomy of the muscles of the ischiotrochanteric group in the albino rat.

Muscle	Origin	Insertion	Innervation
Obturator internus muscle	Inner aspect of tabula of ischium as far as the caudal border of obturator foramen	Conjoined tendon (biceps coxae muscle): Medial aspect of greater trochanter Capsule of hip joint	Nerve to obturator internus muscle (from pudendal nerve, or its branches, or from sacral plexus)
Gemellus muscle	Cranial portion: lesser sciatic notch around tendon of the obturator internus muscle Caudal portion: unnamed groove dorsal on the ischium cranial to ischial tuber		Nerve to quadratus femoris muscle (from sciatic nerve)
Quadratus femoris muscle	Outer aspect of tabula of ischium	Distal portion of intertrochanteric crest Dorsal aspect of lesser trochanter	Nerve to quadratus femoris muscle (from sciatic nerve)

For each muscle, the exact origin, insertion, and innervating nerves are summarized here.

### Obturator internus and gemellus muscles—biceps coxae muscle

**Figure 3:**
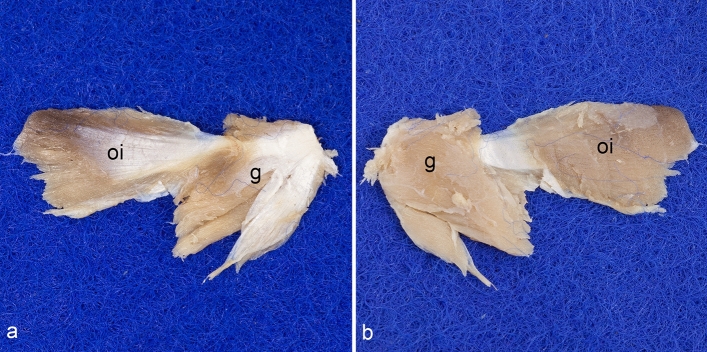
Detached biceps coxae muscle. The entire biceps coxae muscle complex was harvested here. (**a**) Superficially, the gemellus muscle appears to consist of two separate individuals. (**b**) However, on its deep aspect it becomes clear that it is actually one single muscle, which also forms a continuum with the obturator internus muscle. This whole muscle complex is therefore referred to here as the biceps coxae muscle. g = gemellus muscle, oi = obturator internus muscle.

The obturator internus and gemellus muscles are arranged in series building a common tendon of insertion. The two muscle bellies can be only separated by sharp dissection along their approximate border. Therefore, both muscles together will be termed biceps coxae muscle in this study. The obturator internus muscle forms the intrapelvine portion, and the gemellus muscle the extrapelvine portion of this muscle mass. On the sharp edge of the dorsal border of the ischium caudal to the lesser sciatic notch, they are in close contact. Here, the caudal portion of the obturator internus muscle continues more or less into the caudal portion of the gemellus muscle. Both parts are of approximately the same size, but the gemellus muscle is considerably thicker. From the superficial aspect, the gemellus muscle seems to consist of two separate muscles, a small cranial and a large caudal one. However, on its deep aspect, it is one inseparable individual (Fig. 3).

**Figure 4:**
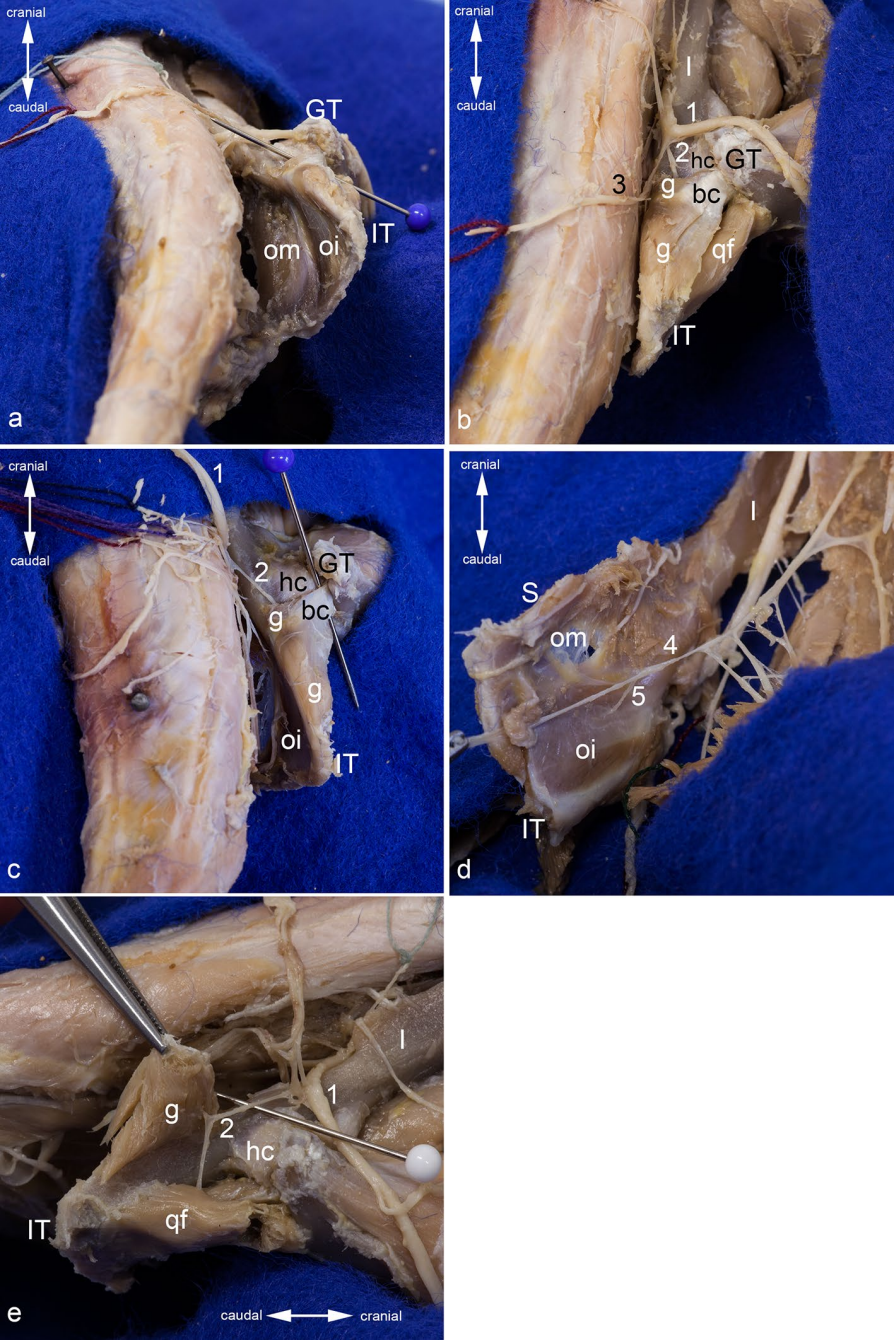
Obturator internus and gemellus muscles. The position of the obturator internus and gemelli muscles is shown (**a**) from caudomedial, (**b**) from dorsal, and (**c**) from dorsomedial. (**d**) The innervation of the obturator internus muscle is shown from ventromedial. (**e**) The innervation of the gemellus muscle is shown from lateral. The muscles of the gluteal, the hamstring, and the adductor groups (except the obturator externus muscle) are detached. The sacroiliac joint is luxated, and the pelvic symphysis cut to get access into the ischiorectal fossa. bc = tendon of biceps coxae muscle, g = gemellus muscle, GT = greater trochanter, hc = hip joint capsule, I = ilium, IT = ischial tuber, oi = obturator internus muscle, om = obturator membrane, qf = quadratus femoris muscle, 1 = sciatic nerve (always dislocated from its true position to gain a complete view), 2 = nerve to quadratus femoris muscle, 3 = superficial perineal nerve, 4 = branch of pudendal nerve, 5 = nerve to obturator internus muscle.

The obturator internus muscle is a pennate muscle, which narrows towards its tendon of insertion. It originates from the inner aspect of the tabula of ischium as far as the caudal border of the obturator foramen (Figs. 2b and 4a). Thus, it is situated caudally to the obturator membrane. The tendon of insertion of the obturator internus muscle courses through the lesser sciatic notch. There, this tendon is deflected in an angle of approximately 90° (Fig. 4a). Therefore, the large sciatic bursa of obturator internus muscle is situated between the deep aspect of this tendon and the bone. The gemellus muscle, which is also a pennate muscle, arises from the lesser sciatic notch around the tendon and the sciatic bursa of obturator internus muscle, and from an unnamed groove, which is located cranially to the ischial tuber (Figs. 2a,c, 4b,c). This groove is separated from the tabula of ischium by a sharp unnamed ridge, which connects the ischial tuber and the acetabulum (Fig. 1e,h). Just outside the lesser sciatic notch, the gemellus muscle fuses with the tendon of the obturator internus muscle, forming the biceps coxae muscle. The tendon of this biceps coxae muscle reinforces the dorsal part of the hip joint capsule, prior to its insertion on the medial aspect of the greater trochanter (Figs. 2d, 4b,c). The obturator internus muscle is innervated by the nerve to obturator internus muscle. It branches off from the pudendal nerve, one of its main branches, or is a direct branch of the sacral plexus. It enters the obturator internus muscle on its superficial surface by piercing its strong fascia (Fig. 4d). The gemellus muscle is innervated by the nerve to quadratus femoris muscle, which enters the muscle on its deep surface (Fig. 4e).

### Quadratus femoris muscle

**Figure 5:**
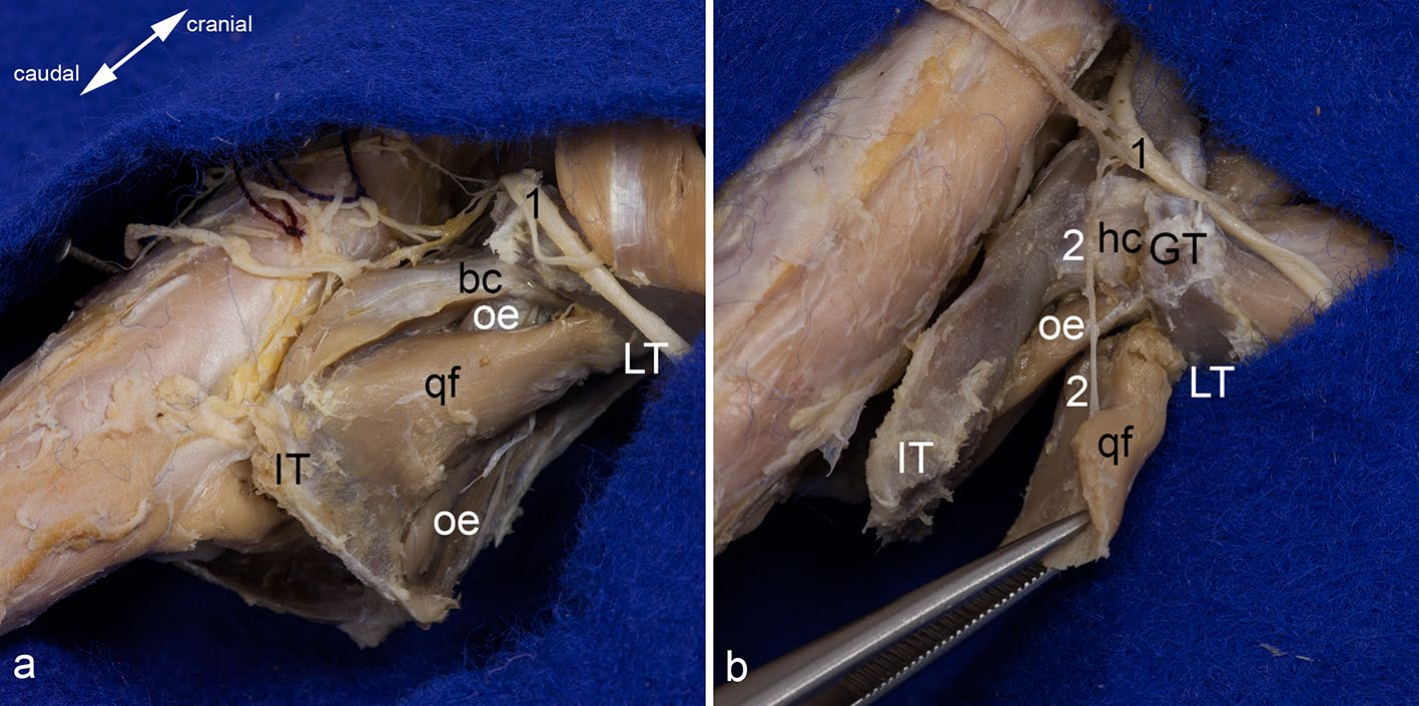
Quadratus femoris muscle. (**a**) Origin and insertion of the quadratus femoris muscle is shown from dorsolateral. (**b**) Its innervation is shown from dorsal. The muscles of the gluteal, the hamstring, and the adductor groups (except the obturator externus muscle) are detached. In (**b**), the sacroiliac joint is luxated, and the pelvic symphysis cut. bc = tendon of biceps coxae muscle, GT = greater trochanter, hc = hip joint capsule, IT = ischial tuber, LT = lesser trochanter, oe = obturator externus muscle, qf = quadratus femoris muscle, 1 = sciatic nerve (always dislocated from its true position to gain a complete view), 2 = nerve to quadratus femoris muscle.

The quadratus femoris muscle is the thickest of this muscle group. It originates from the outer aspect of the tabula of ischium (Figs. 2a,c, and 5a). It crosses the obturator externus and intermedius muscles and inserts on the distal portion of the intertrochanteric crest, and on the dorsal aspect of the lesser trochanter (Figs. 2d and 5a). The quadratus femoris muscle is innervated by the nerve to quadratus femoris muscle branching off from the sciatic nerve. It enters the muscle on its deep surface (Fig. 5b).

### Topography and functional aspects

**Figure 6:**
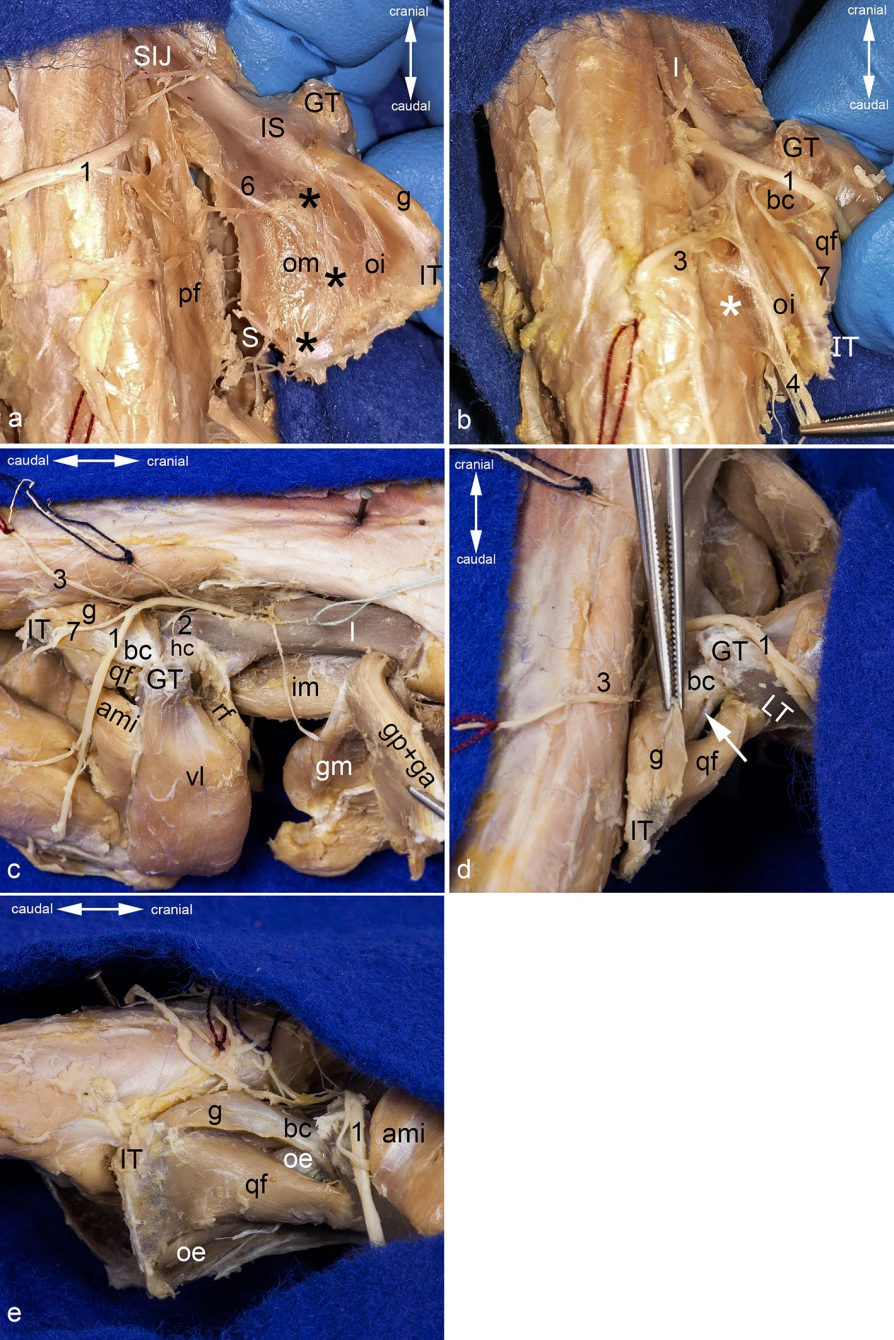
Topography of ischiotrochanteric group. Topographical relationship of the obturator internus muscle. (**a**) The pelvic floor is detached from its origins, the pelvic symphysis is cut, and the sacroiliac joint is luxated. (**b**) The pudendal canal is shown from dorsal. (**c**) Topographical relationship from lateral, (**d**) from dorsal, and (**e**) from caudolateral. The muscles of the gluteal, the hamstring, and the adductor groups (except the obturator externus muscle) are detached at least from their origins. ami = adductor minimus muscle (detached from origin and mobilized), bc = tendon of biceps coxae muscle, g = gemellus muscle, gm = gluteus medius muscle, gp + ga = complex of gluteus profundus and gluteus accessorius muscles, GT = greater trochanter, hc = hip joint capsule, I = ilium, im = iliacus muscle, IS = ischial spine, IT = ischial tuber, LT = lesser trochanter, oe and white arrow = obturator externus muscle, oi = obturator internus muscle, om = obturator membrane, pf = pelvic floor, qf = quadratus femoris muscle, S = pelvic symphysis, SIJ = sacroiliac joint (luxated), black asterisks = tendinous arch of levator ani muscle, white asterisk = external fascia of pelvic diaphragm, 1 = sciatic nerve (dislocated from its true position to gain a complete view in **a**, **d**, **e**), 2 = nerve to quadratus femoris muscle, 3 = superficial perineal nerve, 4 = branches of pudendal nerve, 6 = obturator nerve, 7 = hamstring’s muscular branch.

As described above, the obturator internus muscle originates from the inner aspect of the tabula of ischium caudal to the obturator foramen. In the region of the ischial tuber and the ramus of ischium, it is situated adjacent to the origins of the hamstring and adductor muscles. The obturator internus muscle is covered by a strong fascia. This fascia, together with the obturator membrane, is attached to the dorsocaudal part of the boundary of the obturator foramen. From the junction of these two fascial structures parts of the pelvic floor arise, forming the tendinous arch of levator ani muscle (Fig. 6a). Dorsal to this tendinous reinforcement, the pudendal canal is located between the external fascia of pelvic diaphragm and the fascia of obturator internus muscle. This canal has a craniocaudal course and contains the two main branches of the pudendal nerve and the accompanying vessels (Fig. 6b). The nerve to obturator internus muscle pierces the fascia of the obturator internus muscle to innervate this muscle (Fig. 4d).

The gemellus muscle arises from the ischium around the lesser sciatic notch and from an unnamed groove cranially to the ischial tuber (Figs. 1e,h, 2a,c). There it contacts the origin of the biceps femoris muscle at the dorso-cranial part of the ischial tuber. Its cranial portion has close contact with the gluteus profundus muscle and is partially covered by it. The caudal portion is in close contact with and partially covers the quadratus femoris muscle (Fig. 6c,d). The tendon of the biceps coxae muscle covers the common tendon of the obturator externus and intermedius muscles from dorsal (Fig. 6d). The belly of the gemellus muscle and the tendon of the biceps coxae muscle are covered by the caudal portion of the gluteus medius muscle, the caudofemoralis muscle and the vertebral head of the semitendinosus muscle.

The quadratus femoris muscle originates from the outer surface of the tabula of ischium. There it has close contact with the gemellus, pelvic head of semitendinosus, biceps femoris, semimembranosus and obturator externus muscles that originate around the tabula of ischium. Dorsally, the quadratus femoris muscle is covered proximally by the gemellus and vertebral head of the semitendinosus muscle and subsequently by the caudofemoralis muscle. The ventral portion of the quadratus femoris muscle is flattened near its origin by the course of the large semimembranosus muscle. The adductor minimus muscle superficially crosses the half of the quadratus femoris muscle close to its insertion. The quadratus femoris muscle is oriented nearly horizontally and thus it crosses the obturator externus muscle, which courses diagonally craniodorsal to its insertion in the trochanteric fossa. Therefore, the quadratus femoris muscle has no direct contact with the capsule of the hip joint (Figs. 5a and 6e). In the region of its insertion, the quadratus femoris muscle is in close contact with the insertion of the iliopsoas muscle.

The sciatic nerve runs superficially to the biceps coxae and quadratus femoris muscles. There its tibial part gives off the muscular branch for the hamstring muscles. Still deep to the piriformis muscle, the sciatic nerve gives off the nerve to quadratus femoris. The latter sends a small branch into the caudal portion of the gluteus profundus muscle and then disappears deep to the gemellus muscle (Fig. 6c). There it gives off the innervation for this muscle and finally reaches the deep side of the quadratus femoris muscle (Figs. 4e and 5b). At their origin, the gemellus and quadratus femoris muscles are crossed by the superficial perineal nerve running caudally (Fig. 6c).

Since the rat hip joint is a ball and socket joint, in terms of arthrokinematics, it has three main degrees of freedom. The axes for the main movements are as follows: flexion and extension occur about a transverse axis through the femoral head. Abduction and adduction occur about a sagittal axis through the femoral head. External and internal rotation occurs about a longitudinal axis that passes approximately through the shaft of the femur. Because of the location of the quadratus femoris muscle in the neutral position of the hip joint (Fig. 6c), it courses dorsally and approximately 90° on the axes of rotation. Therefore, it can powerfully externally rotate the hip joint. In addition, it can participate in adduction of the femur in this joint. The biceps coxae muscle also lies dorsal to the axis of rotation (Fig. 4b,c). It, too, should be able to participate in external rotation. Due to its course in relation to the abduction and adduction axis, it assists in abduction of the hip joint. Because of its position close to the fibrous capsule, it should also be able to center the femoral head in the acetabulum.

### Dissection guide

To gain complete access to the muscles of the ischiotrochanteric group, all superficial muscles should be at least partially removed. In any case, the hamstring muscles and the superficial parts of the gluteal muscles must be detached from their origins. The deeper parts of the gluteal group must be detached from their insertions on and around the greater trochanter. To increase the mobility of the hip joint for further preparation, it is also advisable to release the muscles of the adductor group from their origins.

**Figure 7:**
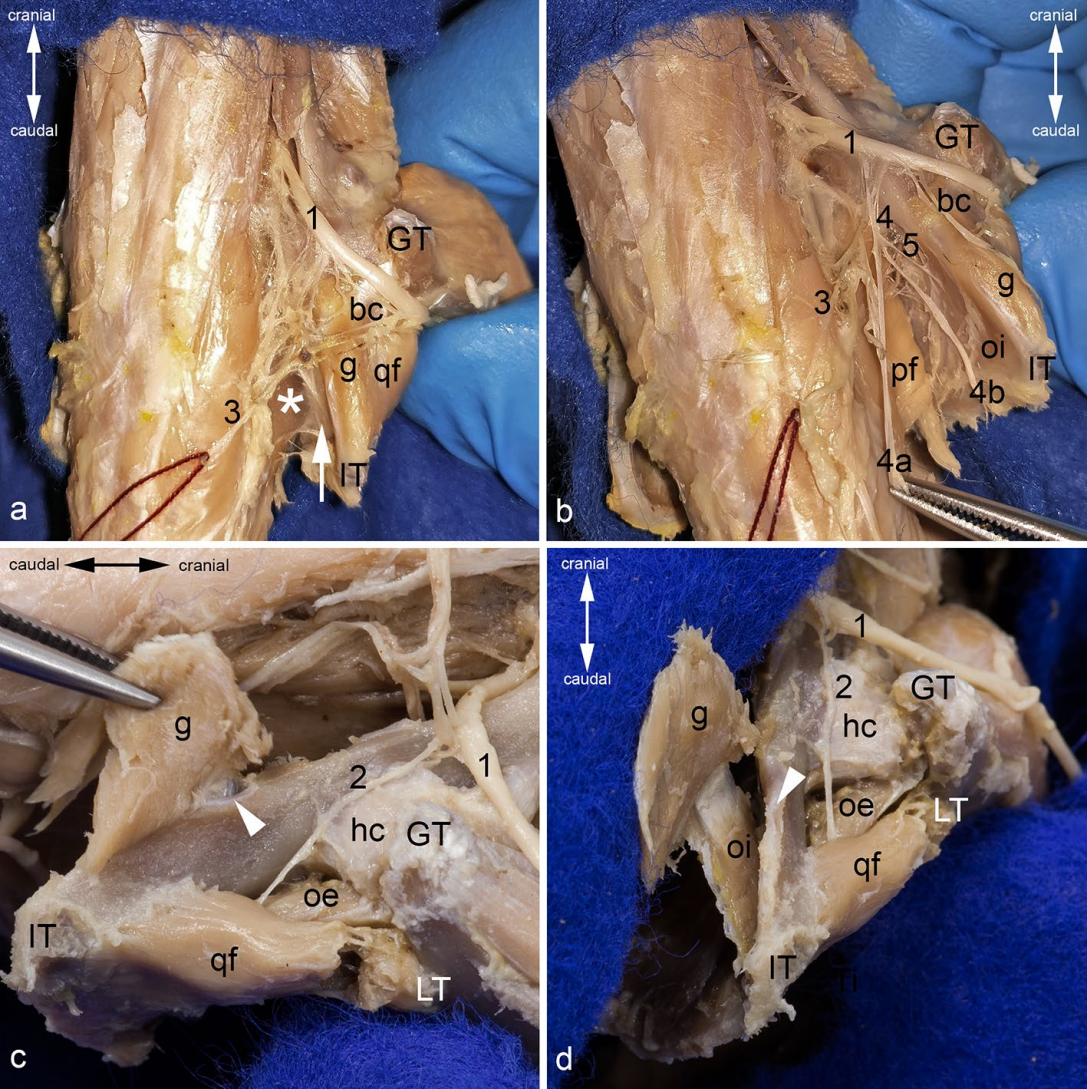
Dissection of the obturator internus muscle and harvesting the biceps coxae musle. (**a**) Approach into the ischiorectal fossa from dorsocaudal. (**b**) Luxation of the sacroiliac joint and opening of the pudenal canal: Both views from dorsal. (**c**) The tendon of the biceps coxae muscle is detached from its insertion, and the gemellus muscle is peeled off from the bone. Its muscular branch from the nerve to quadratus femoris muscle is cut, view from caudolateral. (**d**) The gemellus muscle is completely released, and the obturator internus muscle is partly peeled off from its area of origin, view from dorsal. The muscles of the gluteal, the hamstring, and the adductor groups (except the obturator externus muscle) are detached. The sacroiliac joint is luxated, and the pelvic symphysis cut to get access into the ischiorectal fossa. bc = tendon of biceps coxae muscle, g = gemellus muscle, GT = greater trochanter, hc = hip joint capsule, IT = ischial tuber, LT = lesser trochanter, oe = obturator externus muscle, oi = obturator internus muscle, pf = pelvic floor, qf = quadratus femoris muscle, white arrow = pudendal canal, white arrowhead = sciatic bursa of obturator internus muscle, white asterisk = external fascia of pelvic diaphragm, 1 = sciatic nerve (dislocated from its true position to gain a complete view in **c** and **d**), 2 = nerve to quadratus femoris muscle, 3 = superficial perineal nerve, 4 = pudendal nerve, 4a and 4b = branches of pudendal nerve, 5 = nerve to obturator internus muscle.

The muscle belly of the obturator internus muscle can best be reached via the ischiorectal fossa. To do this, one must widen the gap between the fascia of the muscles of the tail and the hip bone. As there is neither a sacrotuberal nor a sacrospinous ligament in the rat, this is easily possible (Fig. 7a). In order to gain better access, the sacroiliac joint can be luxated carefully and the pelvic symphysis can be cut (Figs. 4d and 6a). Thereby, attention must be paid to the sacral plexus and some of its nerves (sciatic nerve, superficial perineal nerve, nerve to obturator internus muscle, and pudendal nerve) (Fig. 6b). The external fascia of pelvic diaphragm becomes visible, and must be carefully removed by blunt dissection. During this step, the pudendal canal, which is covered by this fascia, is opened and the innervation of the obturator internus muscle can be visualized (Fig. 7b). To gain more space, the pelvic floor can be detached from the tendinous arch of levator ani muscle and from the pubis (Figs. 4d and 6a). During this, it is essential to preserve the innervation of the obturator internus muscle and the obturator nerve (from the lumbar plexus) on its course towards the obturator canal (Fig. 6a).

Harvesting the biceps coxae muscle can be done best by first detaching its tendon of insertion from the medial aspect of the greater trochanter. Thereby, one has to take care of the quadratus femoris muscle and the tendon of the obturator externus muscle, as they are situated close to the biceps coxae muscle. The tendon of the biceps coxae muscle can be carefully detached from its attachments and can be mobilized together with the gemellus muscle towards the lesser sciatic notch. Thereby, the gemellus muscle can be peeled off from the bone by blunt dissection. It is firmly fixed only on the dorsal border of the ischium around the lesser sciatic notch, from which it has to be detached by sharp dissection (Fig. 7c). During mobilization of the gemellus muscle, its innervation by a branch of the nerve to quadratus femoris muscle has to be cut through. The tendon of the obturator internus muscle can now be carefully released from the lesser sciatic notch. Thereby, the sciatic bursa of obturator internus muscle between the bone and the deep aspect of the tendon is opened (Fig. 7c). The obturator internus muscle can be carefully peeled out from the concave inner aspect of the tabula of ischium. Only the borders of the muscle have to be sharply detached from the bone (Fig. 7d).

The quadratus femoris muscle can be detached starting at its insertion on the caudal aspect of the lesser trochanter and the intertrochanteric crest. There it is in close contact to the iliopsoas muscle whose tendon is attached to the ventral part of the lesser trochanter, which should not be injured when mobilizing the quadratus femoris muscle. The latter can be released from its origin on the outer aspect of the tabula of ischium by blunt dissection; during this, the nerve to quadratus femoris muscle has to be cut. When releasing the quadratus femoris muscle, care must also be taken to the obturator externus muscle, which is situated deep to it.

## Discussion

The gemelli muscles and the obturator internus muscle are regarded as three individuals, both in veterinary anatomy^5,22,23,27,28^ and in human anatomy^26^. The rats studied here had one single gemellus muscle, which in addition forms one unit with the obturator internus muscle. This complex is called biceps coxae muscle in this study. The gemelli muscles are said to form two individual muscles in humans, monkeys, many rodents, and other species. In prosimians, insectivores and others they are more or less fused or form a common mass^4^. However, it is often described that the two gemelli muscles—at least deep to the tendon of the obturator internus muscle—are actually one entity^6,8,14,20,29^, as was also consistently observed in this study. Furthermore, the obturator internus and gemelli muscles are frequently considered as one single mass^4,6,14,21,29^. Thereby, the gemellus muscle represents the extrapelvic or accessory head of the obturator internus muscle^6,29^ or, conversely, the obturator internus muscle forms the middle head of the gemelli muscles, which has migrated into the pelvis^14^. The gemelli muscles form a kind of bed or tunnel for the tendon of the obturator internus muscle^7,14^. These three muscles are therefore often referred to as the triceps coxae muscle in humans, at least because of their common insertion^30,31^. The gemelli muscles are subject to wide variation within humans; they may be doubled or even absent. They can be completely fused with the obturator internus muscle itself but also with the quadratus femoris muscle. The latter can even be completely replaced by the gemellus inferior muscle^32^. However, our long experience in the dissection room shows that these variations are extremely rare. In the rats examined here, the caudal part of the obturator internus muscle continued more or less directly into the caudal part of the gemellus muscle. It therefore makes sense to consider the gemelli muscle as one—the extrapelvic—head and the obturator internus muscle as the other—intrapelvic—head of a common muscle mass. This can also be supported by the phylogeny and ontogeny of this muscle group. The muscles of this group originate from a common muscle mass, the quadratus femoris primordialis of the amphibians (anurans)^21^. Only through further developmental steps does the obturator internus muscle separate together with the gemellus muscle from the later quadratus femoris muscle. The obturator internus muscle then gradually migrates to the wall of the lesser pelvis and the gemellus muscle remains in its original position^3,4,21^, apparently without separating completely. This also explains the variations in the appearance of these muscles described above. Therefore, it makes generally more sense to refer to the gemellus muscle (as one individual) as the extrapelvic and the obturator internus muscle as the intrapelvic part of one singular muscle.

In the rats studied here, the obturator internus muscle constantly arose exclusively from the inner aspect of the tabula of ischium, which has never been described before for this muscle. In other rodents (Cricetidae, Heteromyidae), the obturator internus muscle originates either variably from the bones bordering the obturator foramen and the obturator membrane or also from the tabula of ischium (without mentioning this term). In the latter case, however, it additionally arises from the dorsocaudal half of the obturator membrane^5,27^. Parsons^33,34^ studied many different rodents and described the origin of the obturator internus muscle as similar to that of humans, i.e. around the obturator foramen. In rhesus monkeys, it also arises from the inner surface of the pelvis and covers the obturator membrane^8^. In domestic animals, the obturator internus muscle arises around the obturator foramen and possibly also from the sacral bone and from the obturator membrane^4,20,35^. In cloven hoofed animals (artiodactyls), it does not course through the lesser sciatic notch as in the others, but through the obturator foramen^4,20^ and is innervated by the obturator nerve^20^. Looking at the development of this muscle group (see above), it is very doubtful that it is actually an analogous structure of the obturator internus muscle in these cases. In humans, the obturator internus muscle originates from the inner surface of the ventrolateral pelvic wall, medial and caudal to the obturator foramen, from the medial half of the obturator membrane, and its own fascia. The area of origin is said to extend to the ischial tuberosity and the sacrotuberous ligament^6,7,14,15,29^. It is not subject to much variation; rarely it can be absent or completely divided into two parts^32^. The ischium in the region of the ischial tuber is considerably longer (forming the tabula of ischium) in the rat compared to humans, providing enough space for the entire attachment of the obturator internus muscle. In humans and other species with a less pronounced tabula of ischium, the obturator internus muscle apparently has to attach itself further ventrally in the course of its migration into the pelvis.

As observed in this study, the cranial portion of the rat’s gemellus muscle originated constantly from the lesser sciatic notch around the tendon and the sciatic bursa of obturator internus muscle. The larger caudal portion arose from an unnamed groove cranial to the ischial tuber. In principle, this is consistent with the information given by other authors, even if they have used different terms. Thus, in domestic animals, the dorsal margin of the ischium^20^ or the borders of the lesser sciatic notch^4^ are mentioned. In other rodents, the superior gemellus muscle is said to arise from the dorsal surface or margin of the ischium; the inferior gemellus muscle along the "superior ramus of ischium" or the "dorsal ischial margin" until the ischial tuber^5,27,28^. In humans, the gemelli muscles arise from (the dorsal surface of) the ischial spine and (the upper part of) the ischial tuberosity^7,14,29^. According to Yoo et al.^6^ they are attached to the margins of the lesser sciatic notch, the medial aspect of the ischial tuberosity, and the sacrotuberous ligament.

Although in comparative anatomy the trochanteric fossa is usually given as the insertion of the biceps (triceps) coxae muscle^5,20,27,28,35^, in the rat this tendon is attached to the medial surface of the greater trochanter. This is also how it is usually described in humans^6–8,14,29^. In humans, the tendon of the triceps coxae muscle can also unite with that of the piriformis muscle^6,7,14,15,29^. This was never observed in the rats in this study. Walters et al.^36^ observed that the tendon of the triceps coxae muscle constantly reinforces the capsule of the hip joint in its posterior part. It is mentioned in Gray's Anatomy^7^ that there should be a bursa between the tendon and the capsule. Also in this study, the tendon of the biceps coxae muscle was always attached to the dorsal part of the capsule of the hip joint. Since a small hole was often seen in the capsule of the hip joint after this tendon was detached, a small bursa could also be present here, possibly even communicating with the joint cavity. However, this was not investigated in detail here. Apparently, the common tendon of the biceps (triceps) coxae muscle is likely to reinforce the capsule of the hip joint in a dynamic way in both humans and rats.

According to our findings, the quadratus femoris muscle of the rat is relatively similar to that of other mammals and to that of humans. Interestingly, the differences between the species seem to mainly concern the size of this muscle. Ellenberg^20^ described it as a rather weak muscle in domestic animals. Jouffrey^4^ and Hepburn^37^, on the other hand, characterized it as generally robust or always well developed and its shape as square or triangular. According to this study, the quadratus femoris muscle is in any case relatively strongly developed in the albino rat. This is likely to be the case in other rodents as well, following Parsons^33,34^. Interestingly, the official term tabula of ischium is never used for the origin of the quadratus femoris muscle. The dorsal and external portions of the ischial tuber and the ramus of ischium are often referred to^4,28^. As further proven in this study, the quadratus femoris muscle inserts on the intertrochanteric crest and on the caudal aspect of the lesser trochanter in the rat. Descriptions by other authors are very similar here. Ellenberg^20^ and König^35^, for example, described the caudal surface of the femur near the trochanteric fossa. With Jouffrey^4^, the attachment extends to the lesser trochanter or, rarely, even further distally. Rinker^5^ and Stein^27^ described only the lesser trochanter as the attachment zone in the rodents they studied. In humans, the quadratus femoris muscle may be completely absent and replaced by a large inferior gemellus or obturator internus muscle in rare cases^7,29,32^. It may also be divided at its insertion^32^. The ventral surface or (the upper part of) the outer surface of the ischial tuberosity is named as its origin^7,14,29^. Its attachment is described somewhat variable^6^. Thus, Henle^29^ listed the intertrochanteric crest and the area caudal to it. Frohse and Fränkel^14^, on the other hand, wrote that it is almost never attached to this crest, but is attached approximately in the continuation of the gluteal tuberosity until the tip of the greater trochanter. In Gray's anatomy^7^, a small tubercle on the upper part of the intertrochanteric crest and the bone below is mentioned as its attachment.

The muscles of the ischiotrochanteric group are generally innervated by two nerves, the nerve to obturator internus muscle, and the nerve to quadratus femoris muscle. The two nerves are said to be always connected by extra- and intramuscular anastomoses, and in the rhesus monkey, they occasionally even have a common trunk^8^. In humans, they usually originate from the initial part of the sciatic nerve^38^ or are direct branches of the sacral plexus^7,8^. In most domestic animals, both branches originate from the sciatic nerve^20,22^. In the rats of this study, the nerve to quadratus femoris muscle always originated from the initial part of the sciatic nerve, whereas the nerve to obturator internus muscle variably originated from one of the two branches of the pudendal nerve or directly from the sacral plexus. According to Kikuchi^39^, the nerve to obturator internus muscle can be classified into three types with regard to its origin. In humans, the sciatic nerve type is said to be the most common. In rats, dogs, cats and rabbits, however, the pudendal type is said to be the most common, which we were able to confirm for rats in this study. The transitional type is said to be most common in the guinea pig. In humans, the obturator internus muscle is always innervated by the nerve to obturator internus muscle, the quadratus femoris muscle is always innervated by the nerve to quadratus femoris muscle^7,14^. The innervation of the gemelli muscles, on the other hand, is somewhat variable in humans. The superior gemellus muscle is usually innervated by both nerves^6,8^, often only by the nerve to obturator internus muscle^7,8^ and very rarely only by the nerve to quadratus femoris muscle^8^. The inferior gemellus muscle is mostly innervated by the nerve to quadratus femoris muscle^6–8^ and only occasionally in addition by a branch from the nerve to obturator internus muscle^8^. Frohse and Fränkel^14^, who considered the gemelli muscles as one mass, described that it is only innervated by the nerve to obturator internus muscle. In the rhesus monkey, the gemelli muscles are mostly innervated by the nerve to quadratus femoris muscle and only in a quarter of cases the superior gemellus muscle is additionally innervated by the nerve to obturator internus muscle^8^. In the rats of this study, the innervation pattern was constant; the gemellus muscle was always innervated by the nerve to quadratus femoris muscle alone, although it formed a common muscle mass with the obturator internus muscle.

This study confirms the statement that the general gross anatomical arrangement of these muscles varies very little within mammals, which makes them well comparable between species^4,21^. From a macroscopic point of view, the topographical conditions of this muscle group in the rat are, in total, comparable to those of humans, even though the rat is quadrupedal. However, there are some differences in detail, which are summarized here.

Due to the obturator internus muscle originating more caudally in the rat, this muscle lies exclusively in the lateral wall of the ischiorectal fossa. The origin of the pelvic floor muscles completely separates the obturator internus muscle from the wall of the lesser pelvis. Where in humans the obturator internus muscle forms part of the wall of the lesser pelvis, in rats there is only the very thin obturator membrane and, on its outer side, the obturator externus muscle, which partly originates from it. However, this difference only concerns the relative topographical position to the pelvic floor muscles and not to the hip joint. In any case, the gemellus muscle in the rat consists only of one muscle belly. In humans, the gemelli muscles are fused, at least in the region of insertion, and thus are also more likely to consist of one mass^6,14,29^. However, this should definitely be investigated more closely in humans. There are no real differences between humans and rats in the quadratus femoris muscle. It has a relatively larger area of origin in the rat due to the greater extension of the widened transition from the ischial tuber to the body of the ischium in the formation of the tabula of ischium.

We determined the main functions of the biceps coxae and quadratus femoris muscles using a simple arthrokinematic model. Despite the quadrupedal stance of the rat, our results show that these muscles, situated close to the hip joint fulfill almost the same function in the rat as in humans^7^. Despite the rat's squatting position at rest and in motion, we suggest according to Howell^40^ that it should be able to move the hip joint in a similar way to humans. This author further concluded that leverage should therefore be altered primarily in the long double-jointed muscles, which have their insertion much more distally than in humans^40^. The small muscles very close to the hip joint, especially the ischiotrochanteric group, show no striking topographical differences in humans and rats. In both species, straight-line locomotion in the hip joint primarily involves movement in the sagittal plane^7,40^. The abductors and adductors are likely to assist in keeping the leg on course in both species. Presumably, the short lateral rotators of the hip joint have a proprioceptive function rather than being prime movers^7^. The rat also needs proprioceptive control of the hip joint despite its quadruped stance and even more so when climbing. In addition, the rat cannot rotate the foot per se, but this is functionally necessary. Therefore, it must be able to lateral rotate the entire leg in the hip joint^40^.

None of the rats in this study had a sacrotuberal ligament nor a sacrospinous ligament. As far as domestic animals are considered, absence of the sacrospinous ligament has been reported only in cats and dogs^20,35^. The sacrum in these rats was also not as ossified as in other animals or in humans and the individual vertebrae are therefore much more mobile. This is probably due to the very mobile tail of the rat. Ligaments in this area would probably even be a hindrance. The sacrospinous ligament is probably a remnant of the tail musculature^41,42^. As this study has shown, the topographical relationships of the pudendal nerve or its branches to the coccygeal muscle in the rat correspond to those of the pudendal nerve to the sacrospinous ligament in humans. In humans, the nerve runs around the much more pronounced ischial spine around the sacrospinous ligament to enter the pudendal canal. In the rat, its branches run similarly—but somewhat more directly—around the caudal margin of the coccygeal muscle. Apparently, with increasing reduction of the tail and thus of its musculature, the coccygeal muscle—still very strongly developed in the rat—is likely to transform more and more towards the sacrospinous ligament.

This study was limited on the topographical description and comparison of the ischiotrochanteric muscle group. In future, further morphological and functional studies, e.g. analysis of muscle architecture or muscle spindle density, are required to verify the exact function of these muscles.

## Conclusion

This is the first study that has dealt in detail with the description of the ischiotrochanteric muscle group of the albino rat. In addition to a detailed description of these muscles and a dissection guide, their arrangement in the rat was compared with that described for other species. It could be shown that the muscles of the ischiotrochanteric group in the albino rat are topographically very comparable to those of other mammals including humans due to their location, course, and innervation.
